# Characteristics of Acrylic Produced Additively by 3D Printing in Dentistry: Comparison of Mechanical and Surface Parameters—A Systematic Review with Meta-Analysis of Novel Reports

**DOI:** 10.3390/ma18184409

**Published:** 2025-09-21

**Authors:** Paweł Szymlet, Maciej Jedliński, Wojciech Frąckiewicz, Aleksandra Jankowska, Aleksandra Wdowiak-Szymanik, Ewa Sobolewska

**Affiliations:** 1 Ra-Dent Stomatologia Protetyka, Bolesława Krzywoustego Street 19/5, 70-252 Szczecin, Poland; 2 Department of Interdisciplinary Dentistry, Pomeranian Medical University in Szczecin, Powstańców Wlkp. Avenue 72, 70-111 Szczecin, Poland; 3 Apolonia Dental, Gryfińska Street 62A, 70-772 Szczecin, Poland; 4 PeriDent—Dentistry and Periodontology Center, Przecław 119A, 72-005 Przecław, Poland; 5 Independent Pediatric Dentistry Laboratory, Faculty of Medicine and Dentistry, Pomeranian Medical University in Szczecin, Powstańców Wlkp. Avenue 72, 70-111 Szczecin, Poland; aleksandra.wdowiak.szymanik@pum.edu.pl; 6 Department of Dental Prosthetics, Faculty of Medicine and Dentistry, Pomeranian Medical University in Szczecin, Powstańców Wlkp. Avenue 72, 70-111 Szczecin, Poland

**Keywords:** resins, base resins, acrylic, 3D printing, additive manufacturing, dentures, mechanical parameters, surface parameters

## Abstract

**Background/Objectives:** This systematic review and meta-analysis aimed to compare the mechanical and surface properties of three-dimensional (3D) printed and conventionally polymerized acrylic resins. **Methods:** A comprehensive search of four electronic databases (PubMed, Embase, Scopus, and Web of Science) was conducted to identify in vitro studies evaluating impact strength, elastic modulus, surface hardness, and surface roughness. Study quality was assessed using design-specific evaluation tools. When sufficient homogeneous data were available, a meta-analysis was performed. **Results:** The initial search yielded 942 potentially relevant records. Fifteen studies met the criteria for qualitative synthesis, and 13 were included in the meta-analysis. All studies were in vitro and were rated as having moderate to high methodological quality. **Conclusions:** Although conventional acrylic resins currently demonstrate superior mechanical strength, 3D-printed materials exhibit comparable surface properties and continue to evolve rapidly. Additive manufacturing technologies show promise as a viable and effective alternative for future prosthodontic applications.

## 1. Introduction

Contemporary prosthodontics offers a wide range of therapeutic options for patients with partial or complete edentulism [1]. Despite the increasing popularity of fixed restorations, particularly implant-supported prostheses, removable dentures remain an essential component of dental rehabilitation, especially for patients who are ineligible for alternative solutions due to economic or medical constraints. Poly(methyl methacrylate) (PMMA) continues to be the most commonly utilized material in the production of detachable dentures. This material combines favorable mechanical and esthetic properties with relatively low manufacturing costs, making it both accessible and practical in clinical applications [2].

The rapid development of digital technologies in recent years has introduced new possibilities for the fabrication of prosthetic restorations, enabling the use of computer-aided design and computer-aided manufacturing (CAD/CAM) techniques [3]. Three-dimensional (3D) printing has recently gained considerable attention as a potential alternative to conventional acrylic denture base fabrication methods [4]. However, the adoption of 3D printing in prosthodontics requires that the materials fulfill specific mechanical and surface property criteria, including impact strength, hardness, elasticity, and surface roughness [5]. This systematic review aims to compare the mechanical and surface characteristics of denture bases produced using conventional and digitally driven acrylic processing techniques.

### 1.1. History and Importance of PMMA in Prosthetics

Poly(methyl methacrylate) (PMMA) was introduced into dentistry in the first half of the 20th century and remains the material of choice for the fabrication of removable denture bases [6]. Its widespread use results from a favorable combination of mechanical and esthetic properties with acceptable biocompatibility. PMMA exhibits low density, is easily machinable, and allows for the creation of smooth, highly polishable surfaces, which contributes substantially to patient comfort during denture wear [7]. Over the years, advances in material science have led to numerous modifications aimed at enhancing the durability of PMMA, reducing its water sorption, and improving its resistance to microcracks and deformation.

Traditionally, acrylic dentures were fabricated using a flasking technique with heat-polymerized acrylic resin [8]. Although effective, this process is time-consuming, involves multiple steps, and is susceptible to technical errors, particularly when performed by less experienced dental technicians. To streamline the procedure and reduce production time, cold-polymerized acrylic resins were developed [9], which are now widely used, for example, in the fabrication of partial framework dentures and for denture repairs.

The release of leftover methyl methacrylate monomer, which has cytotoxic and irritating effects on oral tissues, has been a recurring problem with acrylic materials. Modern manufacturing protocols and adherence to manufacturer guidelines have significantly mitigated this issue, and clinically relevant toxicity is now rarely observed.

Currently, there is growing clinical interest in the use of digital technologies—such as milling and three-dimensional (3D) printing-for the fabrication of acrylic dentures [10,11]. Although these methods represent a relatively recent development in prosthodontics, they are evolving rapidly and have become the focus of extensive research and clinical implementation.

### 1.2. Three-Dimensional Printing Technologies in Dental Prosthetics

Additive manufacturing (3D printing) has emerged as an increasingly popular alternative to conventional denture fabrication techniques. Digital light processing (DLP) and stereolithography (SLA) are the two main technologies used in dentistry the most. The photopolymerization of liquid, light-curable resins is the basis for both, though the exposure technique and light source are different. In SLA, polymerization occurs point by point using an ultraviolet (UV) laser, whereas DLP simultaneously cures entire layers with a projected light pattern [12].

The advantages of 3D printing include a substantial reduction in production time, improved precision, and the ability to digitally archive prosthetic designs, enabling rapid reproduction in the event of denture damage or loss. Another compelling benefit of additive manufacturing is the potential to decrease the number of clinical visits and reduce overall production costs due to its shortened fabrication workflow [13].

### 1.3. The Evolution of Acrylic Materials in Digital Technology

The application of acrylic materials in 3D printing has necessitated the development of novel material formulations capable of meeting the requirements of photopolymerization-based manufacturing. Conventional PMMA is not directly suitable for additive manufacturing; therefore, specialized acrylic resins, most commonly based on dimethacrylate monomers [14], have been developed. These materials exhibit functional properties similar to those of traditional denture base resins but are compatible with SLA and DLP technologies. Their development required a careful balance between photoreactivity, dimensional stability, residual monomer toxicity, and the mechanical and esthetic properties of the final product [15].

### 1.4. Importance of Mechanical and Surface Properties

For a material to be effectively utilized in the fabrication of denture bases, it must demonstrate appropriate mechanical properties. Among the most critical are:

- Impact strength—the ability of a material to absorb energy from sudden impacts. Brittle materials exhibit low impact strength, whereas more elastic materials demonstrate higher values [16]. This parameter determines a denture’s resistance to fracture caused by accidental drops or masticatory stresses.
- Surface hardness—the resistance of a solid surface to deformation caused by abrasion or indentation by a harder material. In the context of removable dentures, hardness influences the prosthesis’ resistance to wear and deformation under occlusal forces [17].
- Surface roughness—the presence of microscopic surface irregularities resulting from the manufacturing process. This characteristic is particularly important for acrylic resins. A rough denture base surface promotes the accumulation of pathogenic microorganisms, which can adversely affect surrounding oral tissues. Furthermore, higher surface roughness reduces esthetic quality by facilitating pigment penetration into the porous surface [18].

These parameters are critical for ensuring the durability and functional performance of denture bases, and therefore serve as fundamental benchmarks in evaluating the clinical suitability of novel 3D-printed materials.

### 1.5. Aim of the Study

The objective of this systematic review is to compare the mechanical and surface properties of denture bases fabricated using conventional polymerization techniques with those produced through 3D printing. Specifically, this review seeks to determine whether contemporary additive manufacturing materials and methods can constitute a viable alternative to traditional acrylic denture fabrication in terms of durability, functional performance, and clinical safety.

## 2. Materials and Methods

### 2.1. Search Strategy

**Figure 1:**
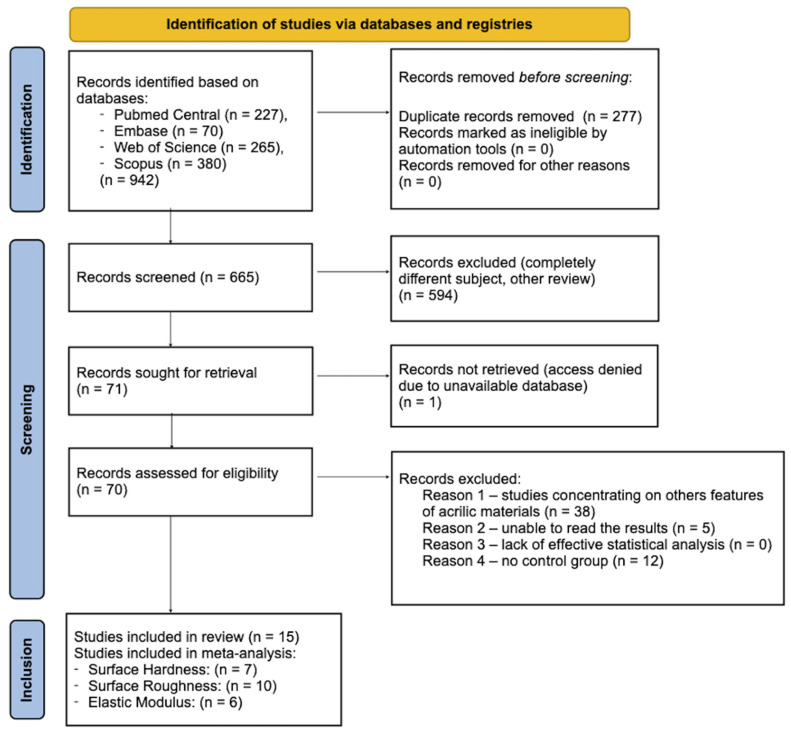
Search strategy—Prisma 2020 flow diagram.

This systematic review was conducted in accordance with the PRISMA statement [19], relevant reporting guidelines [20,21], and the recommendations outlined in the Cochrane Handbook for Systematic Reviews of Interventions [22]. Using the search string (Table S1: see the Supplementary Materials): (resins OR base resins) AND 3D AND print AND denture; initial database searches were started on 10 June 2024, and the final search was conducted on 15 December 2024, spanning PubMed (PMC), Scopus, Web of Science, and Embase. The PRISMA 2020 flow diagram describes minor differences in search syntax between databases (Figure 1).

Following the PICO(S) framework [23], the systematic review was structured as follows:

Population (P): Acrylic resin specimens fabricated using 3D printing techniques;

Intervention (I): Evaluation of mechanical and surface properties;

Comparison (C): Additively manufactured 3D-printed specimens versus conventionally fabricated specimens;

Outcomes (O): Quantitative values of assessed mechanical and surface characteristics;

Study design (S): In vitro studies.

The formulated PICO(S) research question was: “In contemporary dentistry, can 3D-printed acrylic resins serve as a viable alternative to conventionally manufactured materials for denture base fabrication?”.

### 2.2. Eligibility Criteria

The following inclusion criteria were used in this systematic review:

Type of study: in vitro studies;
Outcome measures: surface properties such as surface roughness, and mechanical properties including hardness, elasticity, impact strength, and elastic modulus;
Subject of investigation: evaluation of the surface and mechanical characteristics of acrylic materials made via 3D printing in contrast to traditional techniques;
Material focus: acrylic materials.

The exclusion criteria were as follows:

Studies not related to acrylic resin design;
Retrospective in vivo studies;
Ex vivo studies employing finite element analysis and computer simulations;
Case reports, reviews, expert opinions, book chapters, and conference abstracts;
Studies lacking appropriate statistical analysis;
Studies not related to subtractive manufacturing but involving other acrylic fabrication technologies.

No language restrictions were applied during the search.

### 2.3. Data Extraction

Duplicate articles were eliminated and retrieved. Two authors (P.S. and W.F.) independently assessed the remaining studies based on predetermined eligibility criteria. Full-text articles were carefully reviewed to ensure their suitability for inclusion in this systematic review. In cases of ambiguous results, discussions with a third author (M.J.) helped to address the differences. by jointly analyzing the respective study. When consensus was reached among all three authors, the study was included following the guidelines set forth by the Cochrane Collaboration [22].

Articles discussing the mechanical and surface characteristics of materials made with additive manufacturing—more especially, 3D printing—versus traditional methods were the main focus of the search. Extracted data included quantitative values of the assessed mechanical and surface properties, prioritizing those parameters most frequently reported to enable effective comparison. Information regarding authorship, year of publication, study design, and obtained results related to mechanical and surface parameters was independently extracted by the same reviewers.

### 2.4. Data Synthesis

In the data synthesis, emphasis was placed on extracting information regarding specific mechanical and surface properties—namely surface hardness and roughness, elastic modulus, and impact strength. SI units were used to quantify quantitative data as mean ± standard deviation (SD) (e.g., Vickers Hardness Number [VHN], megapascals [MPa], micrometers [µm], kilojoules per square meter [kJ/m^2^]) in accordance with the original reporting of the analyzed studies.

Where different studies reported results using varying units, conversions were performed to standardize the data, enabling direct comparison. Whenever possible, pooled data were presented in tabular form.

Due to the limited amount of data related to impact strength, a narrative synthesis was employed for this parameter instead of a meta-analysis. The analysis focused on a qualitative comparison of the results, identifying common trends and discrepancies across studies.

### 2.5. Quality Assessment

The Quality Assessment Tool for In Vitro Studies performed in Dentistry (QUIN Tool), a study-type-specific quality assessment scale, was used to assess the risk of bias in the included studies [24]. The QUIN Tool was chosen because it met the search requirements and was pertinent to dental in vitro research, guaranteeing a high degree of accuracy in bias assessment. Two authors (P.S. and M.J.) independently assessed each study using 12 predefined criteria (clearly stated objectives, detailed explanation of sample size calculation, detailed explanation of sampling technique, details of a comparison group, detailed explanation of methodology, operator details, randomization, method of measurement of outcome, outcome assessor details, blinding, statistical analysis, presentation of results). Each criterion was scored as follows: 2 points if adequately described, 1 point if inadequately described, and 0 points if not described. The total score for each study was then calculated and used to classify the risk of bias as low (>70% of points possible to obtain), medium (50–70%), or high (<50%).

### 2.6. Meta-Analysis

The R statistical software version 4.4.2 [25] was used to do meta-analysis utilizing a random-effect model through the metafor R package Base R graphics [26,27]. As an effect estimate, the mean difference (MD) was computed. I2-statistics and Cochran’s Q were used to quantitatively evaluate heterogeneity [28]. The findings were deemed statistically significant when *p* < 0.05. Using a funnel plot and Egger’s test of its asymmetry, publication bias was estimated [29].

## 3. Results

### 3.1. Search Results

Initially, 942 possible articles were found using the employed search strategy: 227 from PubMed and PubMed Central, 70 from Embase, 265 from Web of Science, and 380 from Scopus. 665 items were left for screening after 277 duplicates were eliminated. 594 records were then eliminated since they had nothing to do with the review topic.

Of the remaining 71 studies, one article was excluded due to unavailability in online databases. An additional 38 articles were excluded because they focused on properties of acrylic materials unrelated to the scope of this review. Five articles were rejected because their results could not be accurately extracted, as the data were reported exclusively in graphical form. The final exclusion involved 12 studies that did not include the required control group of conventionally fabricated heat-polymerized acrylic resin denture bases; instead, they used pressure- or injection-molded acrylic resins.

Ultimately, 15 studies—all in vitro experiments—met the inclusion criteria and were analyzed. Figure 1 displays the PRISMA 2020 flow diagram that shows the study selection procedure, and Table 1 provides a summary of the salient features of the selected research. This systematic review was registered on the INPLASY platform (International Platform of Registered Systematic Review and Meta-analysis Protocols) under the registration number INPLASY202580040. The protocol is publicly available at https://doi.org/10.37766/inplasy2025.8.0040 (accessed on 12 August 2025).

### 3.2. Quality Assessment

The risk of bias assessment is presented in the following Table 2 Studies were most frequently lacking the proper sample size calculation, leading to possible lack of significance between the materials tested. Moreover, the studies included were lacking the information regarding the allocation of samples tested and the details of the research team. Some studies failed to provide the results in systematic, tabularized manner or provide full information about the statistical analysis applied.

### 3.3. Meta-Analysis

#### 3.3.1. Meta-Analysis of Studies on Hardness

The first meta-analysis, listed in Table 3, compared the surface hardness of samples additively manufactured using 3D printing and samples manufactured by the canning method using classical acrylic resin. It included 7 articles [30,33,37,38,39,40,42] out of the 15 initially included in Table 1.

In the literature, Vickers microhardness has been reported with different notations such as VHN, HV, or kgf/mm^2^. These designations are equivalent, since the Vickers hardness number (VHN) is defined as the applied load (*F*, in kilograms-force) divided by the lateral surface area of the indentation (*A*, in mm^2^). For clarity and consistency, all hardness values presented in this study were standardized to VHN.

**Figure 2:**
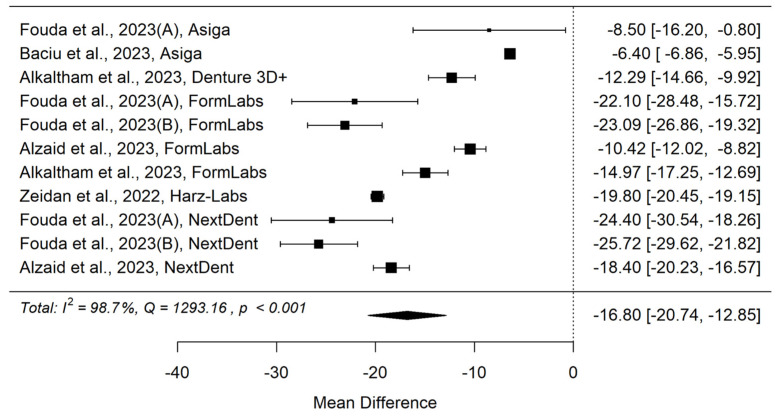
Forest plot for Hardness [33,37,38,39,40,42].

**Figure 3:**
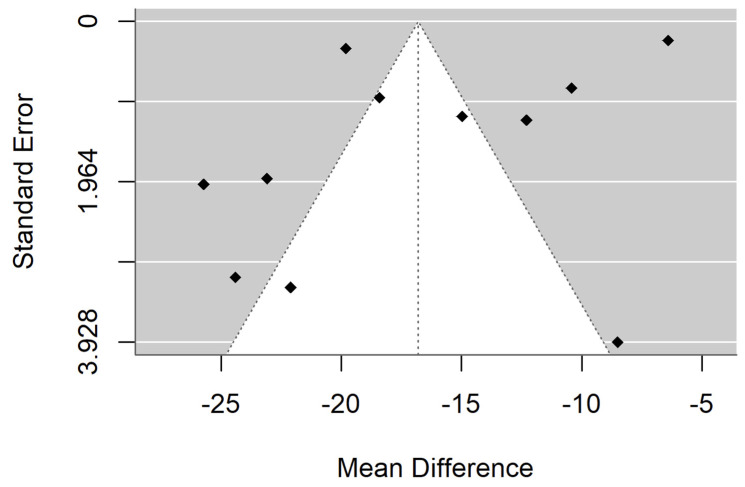
Funnel plot for Hardness.

Figure 2 and Figure 3 show forest plot and funnel plot for Hardness. The Hardness has a large significant (*p* < 0.001) negative effect size. Negative values of effect size mean that Specimens made by Additive Manufacturing have smaller Hardness. Study results are inconsistent—heterogeneity is significant (*p* < 0.001), more than 98% of the variability comes from heterogeneity. Egger’s test indicates funnel plot asymmetry (*p* = 0.024), which can be caused by publication bias.

#### 3.3.2. Meta-Analysis of Studies on Surface Roughness

The second meta-analysis, which included 10 articles [31,32,33,34,38,39,40,41,43,44], aimed to compare the surface roughness of 3D-printed acrylic (additive technology) and that of thermopolymerizing acrylic resin, and its results are presented in Table 4.

Standard deviations in Souza et al., 2024 [44] were calculated basing on confidence intervals using the formula:

$$CI=\underline{m}\pm Z^{*}\frac{\sigma }{\sqrt{n}}$$

where

*CI*—confidence interval

$\underline{m}$—mean value

$Z^{*}$—the critical value of normal distribution

σ—standard deviation

n—the population size

**Figure 4:**
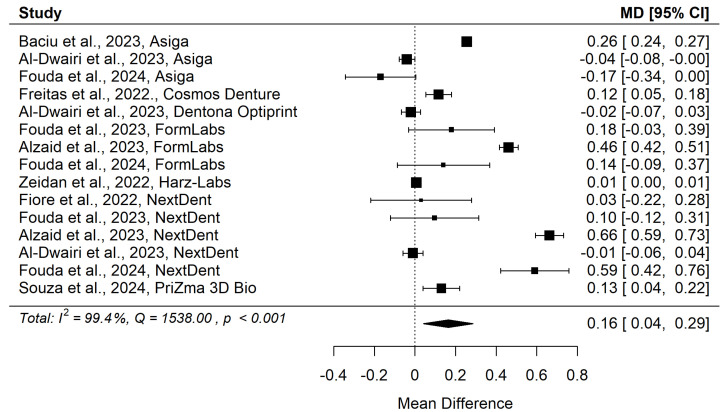
Forest plot for Surface Roughness [31,32,33,37,38,39,40,41,43,44].

**Figure 5:**
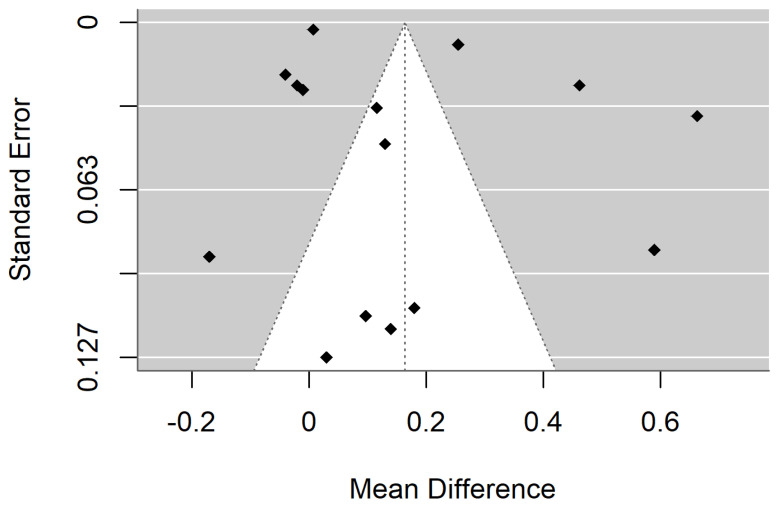
Funnel plot for Surface Roughness.

Figure 4 and Figure 5 show forest plot and funnel plot for Surface Roughness. The Surface Roughness has significant (*p* = 0.009) positive effect size. Positive values of effect size mean that Specimens made by Additive Manufacturing have larger Surface Roughness. Study results are inconsistent—heterogeneity is significant (*p* < 0.001), more than 99% of the variability comes from heterogeneity. Egger’s test does not indicate funnel plot asymmetry (*p* = 0.897).

#### 3.3.3. Meta-Analysis of Studies on Elastic Modulus

The most recent meta-analysis examined the elasticity modulus, with 6 studies [32,33,37,41,42,44] reporting on it in our review. The results of this meta-analysis are presented in Table 5.

**Figure 6:**
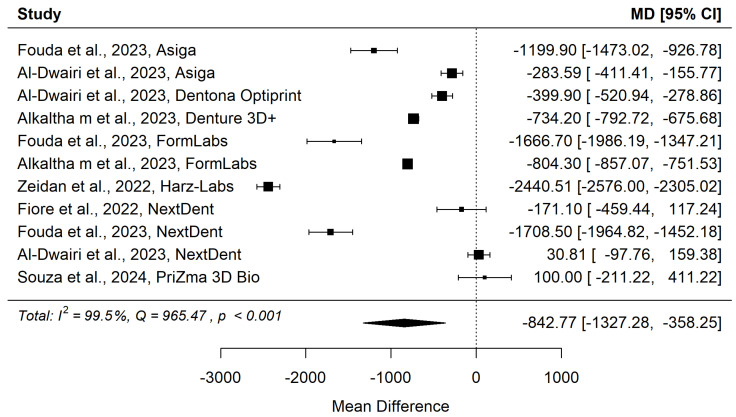
Forest plot for Elastic Modulus [32,33,37,39,41,42,44].

**Figure 7:**
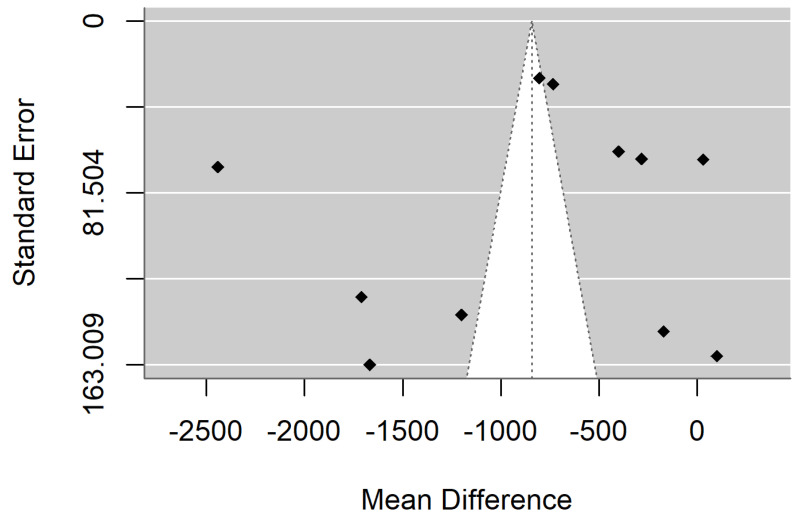
Funnel plot for Elastic Modulus.

Figure 6 and Figure 7 show forest plot and funnel plot for Elastic Modulus. The Elastic Modulus has a large significant (*p* < 0.001) negative effect size. Negative values of effect size mean that Specimens made by Additive Manufacturing have smaller Elastic Modulus. Study results are inconsistent—heterogeneity is significant (*p* < 0.001), more than 100% of the variability comes from heterogeneity. Egger’s test does not indicate funnel plot asymmetry (*p* = 0.813).

## 4. Discussion

Properties like surface roughness, impact strength, surface hardness, and elastic modulus of materials used for denture base production using traditional and 3D printing methods have already been compared in a number of systematic reviews. Before our literature search, the most current review was released in 2022 [45].

Since then, numerous new studies have emerged and were incorporated into the present review. In addition, the literature search was expanded to include the Scopus database. A total of 15 in vitro studies focusing on the mechanical and surface properties of acrylic resins were analyzed. The most frequently evaluated parameters were surface hardness, roughness, and elastic modulus.

Overall, 3D-printed resins demonstrated lower surface hardness compared with conventionally polymerized materials [42], although the ASIGA resin achieved the highest performance among the 3D-printed groups [39].

### 4.1. Technological Changes in Digital Prosthetics

In recent years, dentistry has become one of the primary beneficiaries of the rapid development of 3D printing technologies. The costs of devices have decreased substantially, while their accuracy has improved to a level that allows for the fabrication of definitive prosthetic restorations with high precision [46].

Technologies such as stereolithography (SLA) and digital light processing (DLP) enable rapid and reproducible manufacturing. 3D-printed models are characterized by high dimensional accuracy, reduced operator-dependent errors, and the elimination of direct contact with dust-generating materials, thereby improving laboratory ergonomics [47]. Systems such as ASIGA and Formlabs allow for enhanced anatomical reproduction and minimize polymerization shrinkage, resulting in better adaptation of denture bases to the prosthetic field.

### 4.2. Comparison of Resin Properties

The reviewed studies revealed considerable variability in the mechanical properties of 3D-printed resins depending on the manufacturer. For instance, NextDent resin exhibited the lowest flexural strength and elastic modulus, whereas ASIGA resin consistently demonstrated the highest values among the tested additive materials [39].

Regarding surface hardness, 3D-printed resins generally presented lower values compared to conventional heat-polymerized acrylics. Within the group of printed resins, ASIGA again achieved the most favorable parameters, while the differences between Formlabs and NextDent resins were not statistically significant [39].

#### 4.2.1. Mechanical Parameters

3D printed samples, especially those made from HARZ-Labs and NextDent resins, were characterized by lower hardness than conventional materials [33]. The best results in this group were obtained with ASIGA resin [39]. Printed resins, especially NextDent and HARZ-Labs, showed lower modulus of elasticity and flexural strength [33]. Thermopolymerized acrylic resins showed better flexural strength and impact resistance than their 3D printed counterparts [36]. Although the properties of additive materials are considered clinically acceptable, their limitations in terms of mechanical resistance mean that they are currently recommended primarily for temporary prostheses [38].

#### 4.2.2. Surface Roughness

Fiore et al. [32] used a polishing protocol involving a silicone rubber polishing bur and pumice paste, applied without water, which allowed for the assessment of surface roughness profile (R-profile) before and after polishing. Çakmak et al. [34] described a two-step polishing procedure utilizing an aqueous pumice slurry followed by Fabulustre polishing paste, achieving final Ra values below 0.2 µm. Baciu et al. [38] implemented a more complex, multi-stage protocol that involved different grits of AcryPoint instruments, followed by polishing with a cloth wheel and polishing paste, resulting in a high-gloss finish.

Other studies, including those by Funda et al. [39], Alzaid [40], Al-Dwairi [41], Fouda [43], and Souza [44], primarily employed silicon carbide abrasive papers (typically 1200-grit), sometimes in combination with polishing pastes or other mechanical methods, often under wet conditions. These methods were aimed at surface standardization and were frequently performed by a single operator to reduce procedural variability.

It is worth noting that although the polishing procedures differed significantly in their specific parameters (e.g., duration, applied pressure, type of polishing paste, presence of cooling medium), most studies reported a substantial reduction in surface roughness post-polishing, regardless of the base material. In some cases (e.g., Funda et al. [39]), the durability of the polishing effect was also evaluated through simulated brushing, providing insights into the clinical relevance of surface treatments.

Surface roughness is a significant parameter influencing plaque retention and the aesthetics of restorations. Studies have shown that ASIGA and Formlabs resins did not differ significantly from conventional materials, while NextDent and HARZ-Labs achieved significantly higher roughness values [33,43]. In a study by Al-Dwairi et al. (2022), ASIGA resin demonstrated the lowest roughness among all groups analyzed, including conventional materials [41].

The collected data suggest that polishing protocols have a significant impact on the final surface roughness and should be carefully considered when designing studies and interpreting results. The lack of standardized polishing methods across prosthetic material studies may contribute to discrepancies in reported outcomes.

#### 4.2.3. Environmental Factors

According to Alkaltham et al. [42], various disinfectants exert a significant influence on the hardness, elastic modulus, and flexural strength of denture base materials. The most detrimental effect was observed for sodium hypochlorite (NaOCl).

Furthermore, it has been demonstrated that saliva pH can negatively affect the mechanical properties of both conventional and 3D-printed resins. Immersion of specimens in saliva with fluctuating pH levels resulted in a decrease in surface hardness and flexural strength [40].

### 4.3. Cost-Effectiveness

The implementation of 3D printing involves initial costs, including the purchase of printers, staff training, and dedicated software. However, in the long term, it can provide substantial economic benefits [48]. Automation, a reduction in human error, digital archiving of designs, and shorter production times translate into increased efficiency and competitiveness for dental practices and prosthetic laboratories [49].

### 4.4. Limitations of the Study

The overall quality of the studies can be considered medium [30,31,32,34,35,36,37,38,40,42,43,44] and high (low risk of bias) [33,39,41] so most of the included studies present a level of quality which allows to suppose that given characteristics may also be present in clinical trial. Despite the broad scope of the analysis, this review has several important limitations:

All studies were conducted in vitro—no real clinical validation.

- There is significant methodological heterogeneity: different printers, exposure settings, post-processing protocols, and sample storage conditions.
- The limited number of studies for some resins makes it difficult to generalize the results.
- There is a lack of data on the long-term aging of resins, their resistance to biofilm, discoloration, and wear and tear.
- The lack of clinical trials with patients limits the ability to clearly assess the effectiveness and durability of 3D printed restorations as long-term solutions.

Meta-analyses of hardness, surface roughness, and elastic modulus revealed very high heterogeneity (I^2^ > 99%). Subgroup analyses were considered, taking into account factors such as material brand, printing technology (DLP vs. SLA), and post-processing procedures. However, most primary studies did not provide sufficiently detailed and consistent data in this regard, preventing such analyses. This highlights the need for more systematic reporting of key methodological aspects in future studies to enable more accurate and comparable assessments.

### 4.5. Hopes for the Future of 3D Printed Resins

Technological advancements in 3D printing are transforming the field of prosthodontics. Modern techniques such as SLA, DLP, and proprietary systems like LFS (Formlabs, Somerville, MA, USA) allow for highly precise reproduction of anatomical structures with minimal human error. The use of 3D printing also reduces polymerization shrinkage and residual monomer content, resulting in improved adaptation of the denture base and enhanced patient safety. Future developments in photopolymer chemistry—aimed at increasing elasticity, impact resistance, and long-term durability—combined with the implementation of multi-material printing are expected to enable the production of fully functional, long-term prosthetic solutions rather than temporary restorations alone.

## 5. Conclusions

1. Conventional resins still outperform most 3D printed materials in terms of hardness, flexural strength, and elastic modulus.
2. Some additively printed resins, particularly ASIGA, exhibit properties similar to conventional materials and even superior in terms of surface roughness.
3. 3D-printed resins are promising for selective clinical applications but not yet equivalent to conventional heat-polymerized materials.
4. The cost of implementing 3D printing technology in dental offices and laboratories is initially high, but over time it can become cost-effective thanks to automation, error reduction, and a faster production process.
5. 3D printed prostheses may currently be considered a temporary solution, but given the pace of technological and material development, they can be expected to become a fully fledged alternative to traditional conventional methods in the future.

## Figures and Tables

**Table 1:** Characteristics of included studies.

Author and Year	Type of Article	Material or Subjects	Control Sample or Group	Method	Outcome Measured	Results
Prpić et al., 2020 [30]	In vitro experimental study	Additively manufactured (AM)/3D-printed: 1. 10 samples of dimension 64 × 10 × 3.3 ± 0.2 mm Material: NextDent Base (Nextdent B.V., Soesterberg Netherlands) Printer: DentalFab (Microlay Dental 3D Printers, Madrid, Spain) (DLP)	Heat Polymerizing Acrylic resin: 1. 30 samples of dimension 64 × 10 × 3.3 ± 0.2 mm Material: - ProBase Hot (Ivoclar Vivadent AG, Schaan, Liechtenstein) - Paladon 65 (Kulzer GmbH, Hanau, Germany) - Interacryl Hot (Interdent d.o.o., Celje, Słowenia)	Surface hardness: testing with Zwick apparatus (Zwick Härteprüfgerät Modell 3106 No. 29542/1965, Zwick Roell Group, Kennesaw, GA, USA)	Surface hardness was determined using Brinell’s method, which involved applying stress with a ball for 60 s and measuring Brinell’s hardness at five different locations on each specimen.	Surface hardness [MPa]: 1. 3D-printer: 123.19 2. Heat Polymerizing Acrylic resin: (a) ProBase Hot: 138.13 (b) Paladon 65 123.19 (c) Interacryl Hot 134.06
Freitas et al., 2022 [31]	In vitro experimental study	Additively manufactured (AM)/3D-printed: - 25 disks 10 × 3 mm Material: Cosmos Denture resin (Yller Biomateriais S/A, Pelotas, Brasil) Printer: Hunter (Flashforge, City of Industry, CA, USA) (DLP)	Heat Polymerizing Acrylic resin: - 25 disks 10 × 3 mm Material: Lucitone 199 (Dentsply Sirona, Charlotte, NC, USA)	Surface roughness testing with profilometer SJ-210; Mytutoyo (Corp, Japan).	Surface roughness: Three measurements were taken in the middle of each specimen’s two sides at a 2.0 mm spacing, and the average result was assigned the specimen’s intact Ra value.	Surface roughness [µm]: 1. 3D-printer: - Ra: 0.317 ± 0.151 2. Heat Polymerizing Acrylic resin - Ra: 0.201 ± 0.057
Fiore et al., 2022 [32]	In vitro experimental study	Additively manufactured (AM)/3D-printed: 1. 6 rectangular prism-shaped samples: 65.0 × 10.0 × 3.3 mm Material: Next Dent Denture Base (Nextdent B.V., Soesterberg Netherlands) Printer: MoonRay Model S (VertySystem, Altavilla Vicentina, Italy)	Heat Polymerizing Acrylic resin: 1. 6 rectangular prism-shaped samples: 65.2 × 10.2 × 3.5 mm Material: Aesthetic Blue Clear (Candulor, Rielasingen-Worblingen, Germany)	1. Elastic modulus: Using a universal testing apparatus (Acumen 3; MTS Systems Corp., Eden Prairie, MN, USA), the 3-point bending test was carried out. 2. Surface roughness: Measured using a contact profilometer (Form Talysurf i-Series; Ametek Taylor Hobson, Leicester, UK) both before and after polishing.	1. Elastic modulus: Measured with a crosshead speed of 5 mm/min. Flexural Modulus (MPa) were calculated 2. Surface roughness: In accordance with ISO 16610-21, the Ra was computed using Gaussian filters with the tracing length set to 5.6 mm, the cut-off length set to 0.8 mm, and the stylus speed set to 0.25 mm/s. For every specimen, six measurements were taken. Analysis was performed on the maximum roughness (Rt) and average roughness (Ra). In the provided roughness profile, Ra (µm) is the arithmetic mean value of all heights (peaks and valleys).	1. Elastic modulus [MPa]: (a) 3D-printer: 2371.37 ± 197.30 (b) Heat Polymerizing Acrylic resin: 2542.47 ± 301.55 2. Surface roughness [µm]: (a) 3D-printer: - Ra: 0.38 ± 0.08 (b) Heat Polymerizing Acrylic resin: - Ra: 0.35 ± 0.3
Zeidan et al., 2022 [33]	In vitro experimental study	Additively manufactured (AM)/3D-printed: - 10 samples of dimension 65 × 10 × 3 mm Material: Harz-Labs (Moscow, Russia) Printer: WANHAO-desktop 3D-printer (Zhejiang; China)—DLP	Heat Polymerizing Acrylic resin: - 10 samples of dimension 65 × 10 × 3 mm Material: Vertex-Dental-BV (Soesterberg, The Netherlands)	1. Surface roughness evaluated using an atomic force microscope (Agilent 5600LS AFM, Santa Clara, CA, USA), 2. Elastic modulus testing with three-point bending test (Instron 3345; Buckinghamshire; England) 3. Vickers hardness test (Tukon 1102 Wilson hardness tester, Buehler, Germany) for surface hardness	1. Surface roughness: Five standardized profilometric measurements with an area of 20 × 20 μm^2^ include the AFM (spring constant) and resonance frequency (10 N/m and 250 kHz). 2. Elastic modulus: The load cell is positioned halfway between the two centers of support, with a 50 mm separation and a 5 mm/min crosshead speed. 3. Surface hardness: The specimen was loaded with 300 g, the indenter was held in place for 15 s at three separate points, the focus was achieved using a magnifying eyepiece, and the diagonals of the two prints were measured.	1. Surface roughness [µm]: (a) 3D-printer: - Ra: 0.047 ± 0.00701 (b) Heat Polymerizing Acrylic resin - Ra: 0.03972 ± 0.00472 2. Elastic modulus (MPa): (a) 3D-printer: - E: 576.65 ± 37.73 (b) Heat Polymerizing Acrylic resin - E: 3017.16 ± 215.32 3. Surface hardness (VHN): (a) 3D-printer: 2.64 ± 0.37 (b) Heat Polymerizing Acrylic resin: 22.44 ± 0.98
Çakmak et al., 2022 [34]	In vitro experimental study	Additively manufactured (AM)/3D-printed: - 20 disks 10 mm × 2 mm Material: - Next Dent Denture 3D+ (NextDent B.V., Soesterberg, The Netherlands) - Denturetec (Saremco Dental AG, Rebstein, Switzerland) Printer: - MAX UV (Asiga, Sydney, Australia) (DLP) for Next Dent Denture 3D+ - MoonRay S100 (SprintRay Inc., Los Angeles, CA, USA) (DLP) for Denturetec	Heat Polymerizing Acrylic resin: - 20 disks 10 mm × 2 mm Material: Promolux (Merz Dental GmbH, Lütjenburg, Germany)	Surface roughness: measured using the FRT MicroProf 100 non-contact optical profilometer (Fries Research and Technology GmbH; Bergisch Gladbach, Germany), which has a CWL 300 µm sensor and a 3 nm z-dimension resolution.	Surface roughness: A non-contact optical profilometer was used to capture three parallel line traces spaced 1 mm apart and three parallel line graphs spaced 1 mm apart perpendicular to each other. Each trace measured 5.5 mm in length and had a pixel density of 5501 points per line. Software (Mark III, Fries Research & Technology GmbH; Bergisch Gladbach, Germany) was used to record the initial roughness (Ra), and the average of these traces was computed.	Surface roughness [µm]: 1. 3D-printer: (a) Next Dent Denture 3D+: Ra: 7.95 (b) Denturetec: Ra: 4.18 2. Heat Polymerizing Acrylic resin: Ra: 1.05
Lee et al., 2022 [35]	In vitro experimental study	Additively manufactured (AM)/3D-printed: 1. 25 samples of dimension 64 × 12.7 × 3.2 Material: - Denture Base LP (Formlabs, Somerville, MA, USA) Printer: - Form 2 (Formlabs, Somerville, MA, USA) (SLA)	Heat Polymerizing Acrylic resin: 1. 25 samples of dimension 64 × 12.7 × 3.2 Material: Lucitone 199 (Dentsply Sirona, Charlotte, NC, USA)	Impact strength testing with Izod method (unnotched specimens)	The specimens were marked at the midline, and the impact energy was measured directly from the impact tester in joules. The impact strength was then calculated in kJ/m^2^ using the cross-sectional area of each specimen and an impact strength hinge pendulum.	Impact strength [kj/m^2^] 1. 3D-printer: 11.2 ± 0.7 2. Heat Polymerizing Acrylic resin: 8.9 ± 0.4
Chhabra et al., 2022 [36]	In vitro experimental study	Additively manufactured (AM)/3D-printed: - 15 samples of dimension: 50 mm × 6 mm × 4 mm with a 1.2 mm notch in the middle Material: Next Dent Denture 3D+ (3D Systems, Rock Hill, SC, USA) Printer: NextDent 5100 3D printer (3D Systems, Rock Hill, SC, USA) (SLA)	Heat Polymerizing Acrylic resin: - 15 samples of dimension: 50 mm × 6 mm × 4 mm with a 1.2 mm notch in the middle Material: DPI heat cure, Dental Products of India, (Mumbai, India)	Using a digital Izod/Charpy impact testing machine (International Equipments, Mumbai, India), determine the impact strength.	Impact strength: the specimens were struck by the pendulum at a 150° angle with the notch facing the pendulum hammer (5.4 J). The energy that the specimen absorbed up to the point of fracture was shown on the machine’s digital display.	Impact strength [kJ/m^2^]: (a) 3D-printer: 1.15 ± 0.40 (b) Heat Polymerizing Acrylic resin: 1.67 ± 0.79
Fouda et al., 2023 [37]	In vitro experimental study	Additively manufactured (AM)/3D-printed: 1. 30 samples of dimension: 64 × 10 × 3.3 ± 0.2 mm 2. 30 samples of dimension: 15 × 10 × 2.5 ± 0.2 mm Material: - ASIGA DentaBase, (Asiga pty Ltd., Alexandria, Australia) - FormLabs Denture Base LP (FormLabs, Somerville, MA, USA) - Denture 3D+ (NextDent B.V., Soesterberg, The Netherlands) Printer: - ASIGA Max Printer (DLP) for ASIGA DentaBase, - Form 2 Printer (SLA) for FormLabs Denture Base LP, - NextDent 5100 3D Printer (SLA) for Denture 3D+	Heat Polymerizing Acrylic resin: 1. 10 samples of dimension: 64 × 10 × 3.3 ± 0.2 mm 2. 10 samples of dimension: 15 × 10 × 2.5 ± 0.2 mm Material: - Major.Base.20 (Major Prodotti Dentari, Moncalieri, Italy)	1. Elastic modulus: utilizing a universal testing apparatus (Instron Model 8871; Instron Corp., Canton, MA, USA) to perform a three-point bending test 2. Using a hardness tester (Wilson Hardness; ITW Test And Measurement, GmbH, Shanghai, China) to measure surface hardness	1. Using a universal testing device (Instron Model 8871; Instron Corp., Canton, MA, USA) to measure elastic modulus 2. A Vickers diamond indenter was used to test the surface hardness (25 Gf load for 30 s).	1. Elastic modulus [MPa]: (a) 3D-printer: - ASIGA: 5258.9 (325.9) - FormLabs: 4792.1 (421.6) - NextDent: 4750.3 (288.2) (b) Heat Polymerizing Acrylic resin: 6458.8 (296.6) 2. Surface hardness [VHN]: (a) 3D-printer: - ASIGA: 31.1 (7.5) - FormLabs: 17.5 (2.8) - NextDent: 15.2 (0.15) (b) Heat Polymerizing Acrylic resin: 39.6 (9.9)
Baciu et al., 2023 [38]	In vitro experimental study	Additively manufactured (AM)/3D-printed: 1. 20 samples of dimension: 70 × 30 × 2 mm Material: - Asiga DentaBASE resin (Asiga, Alexandria, NSW, Australia) Printer: - Asiga MAX (Asiga, Alexandria, NSW, Australia) (DLP)	Heat Polymerizing Acrylic resin: 1. 20 samples of dimension: 70 × 30 × 2 mm Material: - Meliodent Heat Cure (Kulzer GmbH, Hanau, Germany)	1. Surface roughness is measured using Form Talysurf, a contact-type roughness tester (Taylor Hobson, Leicester, UK). 2. Surface hardness: Shanghai Daheng Optics and Fine Mechanics Co., Ltd.’s HVT-1000 automatic tester (Shanghai, China) is used for testing.	1. Roughness of the surface Three surface roughness (Ra) measurements were made, and the average of the three measurements was computed and noted. 2. Surface hardness: testing for 10 s with a 50 gf load force. Five conclusions were drawn from each sample.	1. Surface roughness [μm]: (a) 3D-printer: Ra: 0.494 ± 0.028 (b) Heat Polymerizing Acrylic resin: Ra: 0.239 ± 0.024 2. Surface hardness [VHN]: (a) 3D-printer: 13.853 ± 0.586 (b) Heat Polymerizing Acrylic resin: 20.257 ± 0.854
Fouda et al., 2023 [39]	In vitro experimental study	Additively manufactured (AM)/3D-printed: 1. 20 samples of dimension: 12 × 12 × 3 mm Material: - FormLabs shade: light pink (FormLabs, Somerville, MA, USA) - NextDent shade: light pink (NextDent B.V., Soesterberg, The Netherlands) Printer: - Form 2 (FormLabs, Somerville, MA, USA) (SLA) for FormLabs shade: light pink - NextDent 5100 3D system (NextDent B.V., B.V., Soesterberg, The Netherlands) (SLA) for NextDent shade: light pink	Heat Polymerizing Acrylic resin: 1. 20 samples of dimension: 12 × 12 × 3 mm Material: - Major.Base.20 shade: light pink, (Major Prodotti Dentari, Moncalieri, Italy)	1. Surface roughness: measured using a standard camera with a 20× magnification and a profilometer (Contour GT; Bruker Nano GmbH, Schwarzschildstrasse 12, 12489 Berlin, Germany). 2. A hardness tester (Wilson Hardness; ITW Test And Measurement, GmbH, Shanghai, China) was used to measure the surface hardness.	1. Surface roughness: three spots (0.43 × 0.58 μm) on the same side of each specimen were scanned, with three lines on each spot spaced 2 mm apart, and the average value was determined. 2. Surface hardness: determined with a Vickers diamond and a 25-gf stress applied for 30 s.	1. Surface roughness [μm] (a) 3D-printer: - NextDent: Ra: 1.086 ± 0.19 - FormLabs: Ra: 1.169 ± 0.171 (b) Heat Polymerizing Acrylic resin: Ra: 0.989 ± 0.418 2. Surface hardness [VHN]: (a) 3D-printer: - NextDent: 20.57 ± 4.51 - FormLabs: 23.2 ± 4.2 (b) Heat Polymerizing Acrylic resin: 46.29 ± 6.22
Alzaid et al., 2023 [40]	In vitro experimental study	Additively manufactured (AM)/3D-printed: 1. 20 samples of dimension: 15 × 2 × 3.3 ± 0.2 mm Material: - NextDent Denture 3D+ (NextDent, Vertex-Dental B.V, Soesterberg, The Netherlands) - FormLabs denture base LP (FormLabs Inc., Somerville, MA, USA) Printer: - NextDent 5100 (Vertex-Dental B.V, Soesterberg, The Netherlands) (DLP) for NexDent 3D+ - Printer: FormLabs 2 (FormLabs Inc., Somerville, MA, USA) (SLA) for FormLabs denture base LP	Heat Polymerizing Acrylic resin: 1. 10 samples of dimension: 15 × 2 × 3.3 ± 0.2 mm Material: - Major.Base.20 (Major Prodotti Dentari, Moncalieri, Italy)	1. Surface hardness was tested using a blunt indenter with a diameter of 0.8 mm and a load of 300 g for 15 s using a hardness tester (Wilson Hardness; ITW Test And Measurement, GmbH, Shanghai, China). 2. Surface roughness is measured using a noncontact profilometer (Bruker Nano, Tucson, Arizona, United States; Contour Gt-K1 optical profiler).	1. Surface hardness: the average Vickers hardness number (VHN) was determined after testing each specimen five times. 2. Surface roughness: a 0.43 × 0.58 µm area was scanned with a 20× camera. For each specimen, five scans were performed at five distinct locations, and the average was then computed in µm.	1. Surface hardness [VHN]: (a) 3D-printer: - NextDent: 33.83 (2.49) - FormLabs: 41.81 (2.03) (b) Heat Polymerizing Acrylic resin: 52.23 (2.24) 2. Surface roughness [μm]: (a) 3D-printer: - NextDent: Ra: 1.109 (0.094) - FormLabs: Ra: 0.908 (0.044) (b) Heat Polymerizing Acrylic resin: Ra: 0.446 (0.061)
Al-Dwairi et al., 2023 [41]	In vitro experimental study	Additively manufactured (AM)/3D-printed: 1. 45 samples of dimension 25 × 25 × 3 mm 2. 45 samples of dimension 65 × 10 × 3 mm Material: - NextDent Denture 3D+ (NextDent Denture 3D+; Nextdent B.V., Soesterberg, Netherlands) - Dentona Optiprint Denture 3D Printer resin (Dentona Optiprint Denture 3D Printer resin; Dentona AG., Dortmund, Germany) - ASIGA DentaBase (ASIGA DentaBase; ASIGA, Sydney, Australia) Printer ASIGA Max 3D printer (Asiga MAXTM; ASIGA, Sydney, Australia) (DLP)	Heat Polymerizing Acrylic resin: 1. 15 samples of dimension 25 × 25 × 3 mm 2. 15 samples of dimension 65 × 10 × 3 mm Material Meliodent; (Heraeus Kulzer, Hanau, Germany)	1. Using a digital contact profilometer (RT-10; SM S.R.L., Bologna, Italy) to test for surface roughness 2. Elastic modulus A computer-controlled electromechanical universal testing equipment (WDW-20; Jinan Testing Equipment, Jinan, China) is used for 3-point bending testing.	1. Roughness of the surface Surface roughness (Ra) was measured four times, and the average of the four measurements was computed and noted. 2. Elastic modulus A load cell was placed at the specimen’s middle with a crosshead speed of 5 mm/min until fracture, and a 50 mm gap was created between the two centers of support.	1. Surface roughness [µm]: (a) 3D-printer: - NextDent Denture 3D+: Ra: 0.22 ± 0.07 - Dentona Optiprint Denture 3D Printer resin: Ra: 0.21 ± 0.06 - ASIGA DentaBase: Ra: 0.19 ± 0.03 (b) Heat Polymerizing Acrylic resin: Ra: 0.23 ± 0.07 2. Elastic modulus [MPa] (a) 3D-printer: - NextDent Denture 3D+: 2115.80 ± 178.95 - Dentona Optiprint Denture 3D Printer resin: 1685.09 ± 157.14 - ASIGA DentaBase: 1801.40 ± 176.86 (b) Heat Polymerizing Acrylic resin: 2084.99 ± 180.33
Alkaltham et al., 2023 [42]	In vitro experimental study	Additively manufactured (AM)/3D-printed: 1. 40 samples of dimension: 64 × 10 × 3.3 ± 0.2 mm Material: - FormLabs Denture Base LP (FormLabs, Somerville, MA, USA) - Denture 3D+ (NextDent B.V., Soesterberg, The Netherlands) Printer: - FL (FormLabs, Somerville, MA, USA) (SLA) for FormLabs Denture Base LP - NextDent 5100 (3D Systems, Vertex Dental B.V., Soesterberg, The Netherlands) (SLA) for Denture 3D+	Heat Polymerizing Acrylic resin: 1. 20 samples of dimension: 64 × 10 × 3.3 ± 0.2 mm Material: - Major.Base.20 (Major Prodotti Dentari, Moncalieri, Italy)	1. Elastic modulus: utilizing a universal testing apparatus (Instron Model 8871; Instron Corp., Canton, MA) to perform a three-point bending test 2. Vickers hardness test (Wilson Hardness; ITW Test And Measurement GmbH, Shanghai, China) for surface hardness	1. Using a universal testing device (Instron Model 8871; Instron Corp., Canton, MA) to measure elastic modulus 2. Surface hardness: three readings per example were taken at specific locations after each example was placed on the testing apparatus. Every 30 s, a Vickers precious stone indenter with a 25 gf stack was connected. By taking the average of the three readings, the final hardness value of each specimen was determined numerically.	1. Elastic modulus [MPa]: (a) 3D-printer: - FormLabs Denture Base LP: 1144.9 (67.1) - Denture 3D+ 1215.0 (88.5) (b) Heat Polymerizing Acrylic resin: 1949.2 (70.7) 2. Surface hardness [VHN]: (a) 3D-printer: - FormLabs Denture Base LP: 24.25 (2.07) - Denture 3D+ 26.93 (2.32) (b) Heat Polymerizing Acrylic resin: 39.22 (3.04)
Fouda et al., 2024 [43]	In vitro experimental study	Additively manufactured (AM)/3D-printed: 1. 30 samples of dimension: 10 × 12 × 2.5 mm Material: - ASIGA DentaBase, (Asiga Pty Ltd., Alexandria, Australia) - FormLabs Denture Base LP (FormLabs, Somerville, MA, USA) - Denture 3D+ (NextDent B.V., Soesterberg, The Netherlands) Printer: - ASIGA Max Printer (DLP) for ASIGA DentaBase, - Form 2 Printer (SLA) for FormLabs Denture Base LP, - NextDent 5100 3D Printer (SLA) for Denture 3D+	Heat Polymerizing Acrylic resin: 1. 10 samples of dimension: 10 × 12 × 2.5 mm Material: - Major.Base.20 (Major Prodotti Dentari, Moncalieri, Italy)	1. A non-contact optical interferometric profilometer (Contour GT; Bruker Nano GmbH, Berlin, Germany) was used to measure the surface roughness.	1. Surface roughness was measured with a resolution of 0.01 mm in five remote sites. The average Ra value for each sample was then computed.	1. Surface roughness [μm] (a) 3D-printer: - ASIGA: Ra: 0.92 ± 0.23 - FormLabs: Ra: 1.23 ± 0.33 - NextDent: Ra: 1.68 ± 0.22 (b) Heat Polymerizing Acrylic resin: Ra: 1.09 ± 0.16
Brum Souza et al., 2024 [44]	In vitro experimental study	Additively manufactured (AM)/3D-printed: 1. 15 samples of dimension: 65 × 10 × 3.3 mm Material: - PriZma 3D Bio Denture (Makertech Labs, Tatuí, SP, Brazil) Printer: - Phrozen Sonic Mini 4K (Hsinchu, Taiwan) (SLA)	Heat Polymerizing Acrylic resin: 1. 15 samples of dimension: 65 × 10 × 3.3 mm Material: - Fala Thermo Vipi (Vipi)	1. Elastic modulus: Assessed utilizing a universal testing apparatus (EMIC DL, 2000; São José dos Pinhais, PR, Brazil) and the three-point bending monotonic test. 2. Surface roughness: Assessed with a Mitutoyo SJ-410 contact stylus profilometer (Kanagawa, Japan).	1. Elastic modulus: Using support rollers (Ø = 2 mm) 50 mm apart, the specimens were positioned on a particular jig. A third roller (Ø = 2 mm) applied an increasing load at the center of the bar at a crosshead speed of 5 mm/min until fracture occurred. 2. Surface roughness: Six measurements were made with a sampling length of 4 mm and a cut-off (λϲ) of 0.8 mm, three along each of the x and y axis. Each specimen’s arithmetic mean was determined in compliance with ISO 21920-2 (2021).	1. Elastic modulus [MPa]: (a) 3D-printer: - 2390 (2.27–2.52) (b) Heat Polymerizing Acrylic resin: - 2290 (2.01–2.58) 2. Surface roughness [μm]: (a) 3D-printer: Ra: 0.28 (0.19–0.36) (b) Heat Polymerizing Acrylic resin: Ra: 0.15 (0.12–0.18)

**Table 2:** Quality assessment of in vitro studies according to QUIN assessment tool.

Criteria No.	Criteria	Prpić et al., 2020 [30]	Freitas et al., 2022 [31]	Di Fiore et al., 2022 [32]	Zeidan et al., 2022 [33]	Çakmak et al., 2022 [34]	Lee et al., 2022 [35]	Chhabra et al., 2022 [36]	Fouda et al., 2023 [37]	Baciu et al., 2023 [38]	Fouda et al., 2023 [39]	Alzaid et al., 2023 [40]	AL-Dwairi et al., 2023 [41]	Alkaltham et al., 2023 [42]	Fouda et al., 2024 [43]	Brum Souza et al., 2024 [44]
1	Clearly stated aims/objectives	2	2	2	2	2	2	2	2	2	2	2	2	2	2	2
2	Detailed explanation of sample size calculation	0	0	0	2	0	0	0	2	0	2	2	0	2	2	2
3	Detailed explanation of sampling technique	2	2	2	2	2	1	2	2	2	2	2	2	2	2	2
4	Details of comparison group	2	2	2	2	2	2	2	2	2	2	2	2	2	2	2
5	Detailed explanation of methodology	2	2	2	2	2	1	2	2	2	2	2	2	2	2	2
6	Operator details	0	0	0	1	0	0	0	0	0	2	0	2	0	0	0
7	Randomization	0	0	0	0	0	0	0	0	0	0	0	1	0	0	0
8	Method of measurement of outcome	2	2	2	2	2	2	2	2	2	2	2	2	2	2	2
9	Outcome assessor details	0	0	0	1	2	0	0	0	0	0	0	0	0	0	0
10	Blinding	0	0	0	1	0	0	0	0	0	0	0	0	0	0	0
11	Statistical analysis	2	2	2	2	2	2	2	1	2	2	2	2	1	2	2
12	Presentation of results	1	2	2	2	2	2	2	1	2	2	2	2	2	2	2
13	Overall risk of bias	Medium	Medium	Medium	Low	Medium	Medium	Medium	Medium	Medium	Low	Medium	Low	Medium	Medium	Medium

**Table 3:** Differences in surface hardness between the group in which samples were made using the additive manufacturing (AM)/3D printing method and the group in which samples were made using the flasking method.

Author and Year	Hardness—Additive Manufacturing (AM)/3D-printing Group		Hardness—Heat Polymerizing Acrylic resin Group	
	- Number and Shape, - Dimensions, - Material, - Printer of Specimens	Values	- Number and Shape, - Dimensions, - Material, - Milling-Machine of Specimens	Values
Zeidan et al., 2022 [33]	- 10 ingots, - 65 × 10 × 3 mm, - Material: Harz-Labs (Moscow, Russia) - Printer: WANHAO-desktop 3D-printer (Zhejiang; China),	2.64 ± 0.37 VHN	- 10 ingots, - 65 × 10 × 3 mm - Vertex-Dental-BV (Headquarters the Netherlands),	22.44 ± 0.98 VHN
Fouda et al., 2023 [37]	- 30 ingots, - 64 × 10 × 3.3 ± 0.2 mm, - Material: ASIGA DentaBase, (Asiga pty Ltd., Alexandria, Australia), FormLabs Denture Base LP (FormLabs, Somerville, MA, USA), Denture 3D+ (NextDent B.V.,Soesterberg, The Netherlands), - Printer: ASIGA Max Printer, Form 2 Printer, NextDent 5100 3D Printer,	- ASIGA: 31.1 ± 7.5 VHN - FormLabs: 17.5 ± 2.8 VHN - NextDent: 15.2 ± 0.15 VHN	- 10 ingots, - 64 × 10 × 3.3 ± 0.2 mm, - Material: Major.Base.20 (Major Prodotti Dentari, Moncalieri, Italy),	39.6 ± 9.9 VHN
Baciu et al., 2023 [38]	- 20 ingots, - 70 × 30 × 2 mm, - Material: Asiga DentaBASE resin (Asiga, Alexandria, NSW, Australia) - Printer: Asiga MAX (Asiga, Alexandria, NSW, Australia)	13.853 ± 0.586 VHN	- 20 ingots, - 70 × 30 × 2 mm, - Material: Meliodent Heat Cure (Kulzer GmbH, Hanau, Germany)	20.257 ± 0.854 VHN
Fouda et al., 2023 [39]	- 20 ingots, - 12 × 12 × 3 mm, - Material: FormLabs shade (FormLabs, Somerville, MA, USA), NextDent shade (NextDent B.V., Soesterberg, The Netherlands), - Printer: Form 2, NextDent 5100 3D system	- NextDent: 20.57 ± 4.51 VHN - FormLabs: 23.2 ± 4.2 VHN	- 20 ingots, - 12 × 12 × 3 mm, - Material: Major.Base.20 (Major Prodotti Dentari, Moncalieri, Italy)	46.29 ± 6.22 VHN
Alzaid et al., 2023 [40]	- 20 rectangular prism-shaped samples, - 15 × 2 × 3.3 ± 0.2 mm - Material: NextDent Denture 3D+ (NextDent, Vertex-Dental B.V, Soesterberg, The Netherlands), FormLabs denture base LP (FormLabs Inc., Somerville, MA, USA) - Printer: NextDent 5100 (Vertex-Dental B.V, Soesterberg, The Netherlands), FormLabs 2 (FormLabs Inc., Somerville, MA, USA)	- NextDent: 33.83 ± 2.49 VHN - FormLabs: 41.81 ± 2.03 VHN	- 20 rectangular prism-shaped samples, - 15 × 2 × 3.3 ± 0.2 mm - Material: Major.Base.20 (Major Prodotti Dentari, Moncalieri, Italy)	52.23 ± 2.24 VHN
Alkaltha m et al., 2023 [42]	- 20 pieces after breaking ingot samples of dimension: 64 × 10 × 3.3 ± 0.2 mm, - Material: FormLabs Denture Base LP (FormLabs, Somerville, MA, USA), Denture 3D+ (NextDent B.V., Soesterberg, The Netherlands), - Printer: FL (FormLabs, Somerville, MA, USA), NextDent 5100 (3D Systems, Vertex Dental B.V., oesterberg, The Netherlands)	- FormLabs: Denture Base LP: 24.25 ± 2.07 VHN - Denture 3D+: 26.93 ± 2.32 VHN	- 10 pieces after breaking ingot samples of dimension: 64 × 10 × 3.3 ± 0.2 mm - Material: Major.Base.20 (Major Prodotti Dentari, Moncalieri, Italy)	39.22 ± 3.04 VHN

**Table 4:** Differences in surface roughness between the group in which samples were made by additive manufacturing (AM)/3D printing and the group in which samples were made by flasking.

Author and Year	Surface Roughness—Additive Manufacturing (AM)/3D-Printing Group		Surface Roughness—Heat Polymerizing Acrylic resin Group	
	- Number and Shape, - Dimensions, - Material, - Printer of Specimens	Values	- Number and Shape, - Dimensions, - Material, - Milling-Machine of Specimens	Values
Freitas et al., 2022 [31]	- 25 disks, −10 × 3 mm, - Material: Cosmos Denture resin (Yller Biomateriais S/A, Pelotas, Brasil), - Printer: Hunter (Flashforge, City of Industry, CA, USA) (DLP)	0.317 ± 0.151 μm	- 25 disks, −10 × 3 mm, - Material: Lucitone 199 (Dentsply Sirona, Charlotte, NC, USA)	0.201 ± 0.057 μm
Fiore et al., 2022 [32]	- 6 rectangular prism-shaped samples, - 65.0 × 10.0 × 3.3 mm, - Material: Next Dent Denture Base (Nextdent B.V., Soesterberg Netherlands) - Printer: MoonRay Model S (VertySystem, Altavilla Vicentina, Italy)	0.38 ± 0.08 μm	- 6 rectangular prism-shaped samples, - 65.0 × 10.0 × 3.3 mm, - Material: Aesthetic Blue Clear (Candulor, Rielasingen-Worblingen, Germany)	0.35 ± 0.3 μm
Zeidan et al., 2022 [33]	- 10 ingots, - 65 × 10 × 3 mm - Material: Harz-Labs (Moscow, Russia), - Printer: WANHAO-desktop 3D-printer (Zhejiang; China),	0.047 ± 0.00701 μm	- 10 ingots, - 65 × 10 × 3 mm - Material: Vertex-Dental-BV (Headquarters the Netherlands)	0.03972 ± 0.00472 μm
Çakmak et al., 2022 [34]	- 20 disks, - 10 mm × 2 mm, - Material: Next Dent Denture 3D+ (NextDent B.V., Soesterberg, The Netherlands), Denturetec (Saremco Dental AG, Rebstein, Switzerland), - Printer: MAX UV (Asiga, Sydney, Australia), MoonRay S100 (SprintRay Inc., Los Angeles, CA, USA),	- Next Dent Denture 3D+: 7.95 μm - Denturetec: 4.18 μm	- 20 disks, - 10 mm × 2 mm, - Material: Promolux (Merz Dental GmbH, Lütjenburg, Germany)	1.05 μm
Baciu et al. [38], 2023	- 20 ingots, - 70 × 30 × 2 mm, - Material: Asiga DentaBASE resin (Asiga, Alexandria, NSW, Australia), - Printer: Asiga MAX (Asiga, Alexandria, NSW, Australia)	0.494 ± 0.028 μm	- 20 ingots, - 70 × 30 × 2 mm, - Material: Meliodent Heat Cure (Kulzer GmbH, Hanau, Germany)	0.239 ± 0.024 μm
Fouda et al., 2023 [39]	- 20 ingots, - 12 × 12 × 3 mm, - Material: FormLabs shade (FormLabs, Somerville, MA), NextDent (NextDent B.V., Soesterberg, The Netherlands), - Printer: Form 2 (FormLabs), NextDent 5100 3D system (NextDent),	- NextDent: 1.086 ± 0.19 μm - FormLabs: 1.169 ± 0.171 μm	- 20 ingots, - 12 × 12 × 3 mm, - Material: Major.Base.20 shade: light pink, (Major Prodotti Dentari, Moncalieri, Italy)	0.989 ± 0.418 μm
Alzaid et al., 2023 [40]	- 20 ingots, - 15 × 2 × 3.3 ± 0.2 mm, - Material: NextDent Denture 3D+ NextDent, Vertex-Dental B.V, Soesterberg, The Netherlands), FormLabs denture base LP (FormLabs Inc., Somerville, MA, USA), - Printer: NextDent 5100 (Vertex-Dental B.V, Soesterberg, The Netherlands), FormLabs 2 (FormLabs Inc., Somerville, MA, USA)	- NextDent: 1.109 ± 0.094 μm - FormLabs: 0.908 ± 0.044 μm	- 10 ingots, - 15 × 2 × 3.3 ± 0.2 mm, - Material: Major.Base.20 (Major Prodotti Dentari, Moncalieri, Italy)	0.446 ± 0.061 μm
Al-Dwairi et al., [41]	- 45 ingots, - 25 × 25 × 3 mm, - Material: NextDent Denture 3D+ (NextDent Denture 3D+; Nextdent B.V., Soesterberg, Netherlands), Dentona Optiprint Denture 3D Printer resin (Dentona Optiprint Denture 3D Printer resin; Dentona AG., Dortmund, Germany), ASIGA DentaBase (ASIGA DentaBase; ASIGA, Sydney, Australia), - Printer: ASIGA Max 3D printer (Asiga MAXTM; ASIGA, Sydney, Australia)	- NextDent Denture 3D+: 0.22 ± 0.07 μm - Dentona Optiprint Denture 3D Printer resin: 0.21 ± 0.06 μm - ASIGA DentaBase: 0.19 ± 0.03 μm	- 15 ingots, - 25 × 25 × 3 mm, - Material: Meliodent; (Heraeus Kulzer, Hanau, Germany)	0.23 ± 0.07 μm
Fouda et al., 2024 [43]	- 30 ingots, - 10 × 12 × 2.5 mm, - Material: ASIGA DentaBase, (Asiga pty Ltd., Alexandria, Australia), FormLabs Denture Base LP (FormLabs, Somerville, MA, USA), Denture 3D+ (NextDent B.V., Soesterberg, The Netherlands), - Printer: ASIGA Max Printer, Form 2 Printer, NextDent 5100 3D,	- ASIGA: 0.92 ± 0.23 μm - FormLabs: 1.23 ± 0.33 μm - NextDent: 1.68 ± 0.22 μm	- 10 ingots, - 10 × 12 × 2.5 mm, - Material:	1.09 ± 0.16 μm
Souza et al., 2024 [44]	- 15 ingots, - 65 × 10 × 3.3 mm, - Material: PriZma 3D Bio Denture (Makertech Labs, Tatuí, SP, Brazil) - Printer: Phrozen Sonic Mini 4K (Hsinchu, Taiwan)	0.28 ± 0.09 μm	- 15 ingots, - 65 × 10 × 3.3 mm, - Material: Fala Thermo Vipi (Vipi)	0.15 ± 0.03 μm

**Table 5:** Differences in elastic modulus between the additive manufacturing (AM)/3D printing group and the flasking group.

Author and Year	Elastic Modulus—Additive Manufacturing (AM)/3D-Printing Group		Elastic Modulus—Heat Polymerizing Acrylic resin Group	
	- Number and Shape, - Dimensions, - Material, - Printer of Specimens	Values	- Number and Shape, - Dimensions, - Material, - Milling-Machine of Specimens	Values
Fiore et al., 2022 [32]	- 6 rectangular prism-shaped samples, - 65.0 × 10.0 × 3.3 mm, - Material: Next Dent Denture Base, - Printer: MoonRay Model S (VertySystem, Altavilla Vicentina, Italy)	2371.37 ± 197.30 MPa	- 6 rectangular prism-shaped samples, - 65.0 × 10.0 × 3.3 mm, - Material: Aesthetic Blue Clear (Candulor, Rielasingen-Worblingen, Germany))	2542.47 ± 301.55 MPa
Zeidan et al., 2022 [33]	- 10 ingot, - 65 × 10 × 3 mm - Material: Harz-Labs (Moscow, Russia), - Printer: WANHAO-desktop 3D-printer (Zhejiang; China),	576.65 ± 37.73 MPa	- 10 ingot, - 65 × 10 × 3 mm - Material: Vertex-Dental-BV (Headquarters, the Netherlands)	3017.16 ± 215.32 MPa
Fouda et al., 2023 [37]	- 30 ingot, - 15 × 10 × 2.5 ± 0.2 mm, - Material: ASIGA DentaBase, (Asiga pty Ltd., Alexandria, Australia), FormLabs Denture Base LP (FormLabs, Somerville, MA, USA), Denture 3D+ (NextDent B.V.,Soesterberg, The Netherlands), - Printer: ASIGA Max Printer, Form 2 Printer, NextDent 5100 3D Printer,	- ASIGA: 5258.9 ± 325.9 MPa - FormLabs: 4792.1 ± 421.6 MPa - NextDent: 4750.3 ± 288.2 MPa	- 10 ingot, - 15 × 10 × 2.5 ± 0.2 mm, - Material: Major.Base.20 (Major Prodotti Dentari, Moncalieri, Italy),	6458.8 ± 296.6 MPa
Al-Dwairi et al., [41]	- 45 ingot, - 65 × 10 × 3 mm, - Material: NextDent Denture 3D+ (NextDent Denture 3D+; Nextdent B.V., Soesterberg, Netherlands), Dentona Optiprint Denture 3D Printer resin (Dentona Optiprint Denture 3D Printer resin; Dentona AG., Dortmund, Germany), ASIGA DentaBase (ASIGA DentaBase; ASIGA, Sydney, Australia), - Printer: ASIGA Max 3D printer (Asiga MAXTM; ASIGA, Sydney, Australia)	- NextDent Denture 3D+: 2115.80 ± 178.95 MPa - Dentona Optiprint Denture 3D Printer resin: 1685.09 ± 157.14 MPa - ASIGA DentaBase: 1801.40 ± 176.86 MPa	- 15 ingot, - 65 × 10 × 3 mm, - Material: Meliodent; (Heraeus Kulzer, Hanau, Germany)	2084.99 ± 180.33 MPa
Alkaltha m et al., 2023 [42]	- 40 ingot, - 64 × 10 × 3.3 ± 0.2 mm, - Material: FormLabs Denture Base LP (FormLabs, Somerville, MA, USA), Denture 3D+ (NextDent B.V., Soesterberg, The Netherlands), - Printer: FL (FormLabs, Somerville, MA, USA), NextDent 5100 (3D Systems, Vertex Dental B.V., Soesterberg, The Netherlands)	- FormLabs Denture Base LP: 1144.9 ± 67.1 MPa - Denture 3D+ 1215.0 ± 88.5 MPa	- 10 pieces after breaking ingot samples of dimension: 64 × 10 × 3.3 ± 0.2 mm - Material: Major.Base.20 (Major Prodotti Dentari, Moncalieri, Italy)	1949.2 ± 70.7 MPa
Souza et al., 2024 [44]	- 15 ingot, - 65 × 10 × 3.3 mm, - Material:PriZma 3D Bio Denture (Makertech Labs, Tatuí, SP, Brazil) - Printer: Phrozen Sonic Mini 4K (Hsinchu, Taiwan)	2390 MPa	- 15 ingot, - 65 × 10 × 3.3 mm, - Material: Fala Thermo Vipi (Vipi)	2290 MPa

## Data Availability

No new data were created or analyzed in this study. Data sharing is not applicable to this article.

## Supplementary Materials

The following supporting information can be downloaded at https://www.mdpi.com/article/10.3390/ma18184409/s1, Table S1: Search string strategy.

## Abbreviations

The following abbreviations are used in this manuscript:

3D	Three-Dimensional
AM	Additive Manufacturing
CAD/CAM	Computer-Aided Design/Computer-Aided Manufacturing
CI	Confidence Interval
DLP	Digital Light Processing
LFS	Low Force Stereolithography
MD	Mean Deviation
NaOCl	Sodium Hypochlorite
pH	Potential of Hydrogen
PMC	PubMed Central
PMMA	Polymethyl Methacrylate
PRISMA	Preferred Reporting Items for Systematic Reviews and Meta-Analyses
QUIN	Quality Assessment Tool For In Vitro Studies
SD	Standard Deviation
SLA	Stereolithography
