# Supplementary material for: Characteristics of Acrylic Produced Additively by 3D Printing in Dentistry: Comparison of Mechanical and Surface Parameters—A Systematic Review with Meta-Analysis of Novel Reports
Source: Materials (Basel). 2025 Sep 21;18(18):4409. doi: 10.3390/ma18184409 (PMC12472056; doi:10.3390/ma18184409)
Supplement: Supplementary file 1 [file materials-18-04409-s001.zip › materials-3827659-supplementary.pdf]

Supplementary material.

Table S1. Search strategy.

Order	Term
#1	„Resins”
#2	„Base Resins”
#3	„3D”
#4	„Print”
#5	„Denture"
#6	#1 OR #2
#7	#3 AND #4
#8	#5 AND #6 AND #7

Pubmed / PMC:

[1] – search string: ("resin s"[All Fields] OR "resinous"[All Fields] OR "resins, plant"[MeSH Terms] OR ("resins"[All Fields] AND "plant"[All Fields]) OR "plant resins"[All Fields] OR "resin"[All Fields] OR "resins"[All Fields] OR ("base"[All Fields] AND ("resin s"[All Fields] OR "resinous"[All Fields] OR "resins, plant"[MeSH Terms] OR ("resins"[All Fields] AND "plant"[All Fields]) OR "plant resins"[All Fields] OR "resin"[All Fields] OR "resins"[All Fields]))) AND "3D"[All Fields] AND ("printed"[All Fields] OR "printing"[MeSH Terms] OR "printing"[All Fields] OR "print"[All Fields] OR "printings"[All Fields] OR "prints"[All Fields]) AND ("dentural"[All Fields] OR "denture s"[All Fields] OR "dentures"[MeSH Terms] OR "dentures"[All Fields] OR "denture"[All Fields]) - 227 results

Embase:

[2] – search string: ('resins'/exp OR resins OR 'base resins' OR (('base'/exp OR base) AND ('resins'/exp OR resins))) AND ('3d'/exp OR 3d) AND print AND ('denture'/exp OR denture) - 70 results

Web of Science:

[3] – search string: ALL = ((resin OR "base resin") AND ("3d printing" OR "three-dimensional printing") AND (denture OR dentures)) - 265 results

Scopus:

[4] – search string: TITLE-ABS-KEY ((resin OR "base resin") AND ("3d print*" OR "three-dimensional print*") AND (denture OR dentures)) - 380 results
